# Brain connectivity changes associated with episodic recollection decline in aging: A review of fMRI studies

**DOI:** 10.3389/fnagi.2022.1012870

**Published:** 2022-10-25

**Authors:** Selene Cansino

**Affiliations:** Laboratory of NeuroCognition, Faculty of Psychology, National Autonomous University of Mexico, Mexico City, Mexico

**Keywords:** recollection, familiarity, functional connectivity, effective connectivity, task-related brain connectivity

## Abstract

With advancing age, individuals experience a gradual decline in recollection, the ability to retrieve personal experiences accompanied by details, such as temporal and spatial contextual information. Numerous studies have identified several brain regions that exhibit age-related activation differences during recollection tasks. More recently, an increasing number of studies have provided evidence regarding how brain connectivity among the regions supporting recollection contributes to the explanation of recollection deficits in aging. However, brain connectivity evidence has not been examined jointly to provide an integrative view of how these new findings have improved our knowledge of the neurofunctional changes underlying the recollection deficits associated with aging. Therefore, the aim of the present study was to examine functional magnetic resonance imaging (fMRI) studies that employed one of the numerous methods available for analyzing brain connectivity in older adults. Only studies that applied connectivity analysis to data recorded during episodic recollection tasks, either during encoding or retrieval, were assessed. First, the different brain connectivity analysis methods and the information conveyed were briefly described. Then, the brain connectivity findings from the different studies were described and discussed to provide an integrative point of view of how these findings explain the decline in recollection associated with aging. The studies reviewed provide evidence that the hippocampus consistently decreased its connectivity with the parahippocampal gyrus and the posterior cingulate cortex, essential regions of the recollection network, in older adults relative to young adults. In addition, older adults exhibited increased connectivity between the hippocampus and several widespread regions compared to young adults. The increased connectivity was interpreted as brain intensification recourse to overcome recollection decay. Additionally, suggestions for future research in the field are outlined.

## Introduction

Brain connectivity has been extensively analyzed during resting-state conditions and less when the brain is performing a task. The discovery that spontaneous brain connectivity occurred between brain regions that are associated with specific cognitive demands (e.g., Beckmann et al., 2005) or the finding that some of these brain regions decrease their activity when they are engaged in a cognitive task, such as the default mode network (e.g., Raichle et al., 2001), have provided evidence that resting-state connectivity might play an important role in brain integration, a condition that may be important for preserving cognitive functioning in aging (for reviews see Antonenko and Flöel, 2014; Sala-Llonch et al., 2015). However, spontaneous brain connectivity may be a cause or an effect of cognitive functioning. This issue is difficult to solve because these findings are based only on correlational evidence. Thus, the analysis of brain connectivity while the brain is engaged in a specific cognitive task represents a direct opportunity to examine which regions are actually involved in performing the task and how each other interacts to reach the task goal under conditions of both success and failure. This information is crucial to investigate topics such as age-related cognitive decay, which requires a more detailed explanation to provide or suggest possible solutions. This is particularly important for the case of episodic memory, the type of memory that is considered to be the most affected by the aging process (Nyberg et al., 2012).

Episodic memory is the ability to remember our personal past (Tulving, 1972). Because each of our experiences occurred in a specific context, the capability to retrieve contextual details, such as the location, moment or emotion that accompanied the experience, is conceived as truly episodic and evidence that the memory has been retrieved by means of recollection. This process has been distinguished from familiarity, the ability to remember personal events that lack any contextual detail and provide only a vague awareness that the experience has previously occurred (Mandler, 1980). Moreover, consistent evidence indicates that only recollection is severely affected with advancing age, whereas familiarity is only slightly diminished (Spencer and Raz, 1995; e.g., Cansino et al., 2013). Therefore, to investigate how brain connectivity during episodic memory might be modified in older adults, it is essential to examine studies that reliably assess recollection. For example, the old/new task that is frequently used to examine episodic memory does not allow disentangling whether correct old items had been identified by means of recollection or familiarity. Thus, studies that employed this task will not be included in the present review. Therefore, only functional magnetic resonance imaging (fMRI) studies that examined brain connectivity while participants were engaged in a recollection task were reviewed.

Several brain connectivity analyses have been developed that provide distinctive and a wide range of information on how the brain functions as a network. For a detailed description of these analyses, see Friston (2011). A brief description of the brain connectivity analyses more frequently used is provided below, and most of the methods were employed by the studies reviewed here. Studies of brain structural connectivity, which refers to the neural anatomical connection within the brain, known as the connectome (Sporns et al., 2005), will not be discussed in the current review. Brain connectivity analytic methods are able to measure functional connectivity or effective connectivity. The former measures the dependency between neural events using statistical tests, such as correlation, covariance or coherence. Conversely, effective connectivity provides evidence of the influence that a neural unit, either unicellular or multicellular, exerts over other units within the neural system under examination (Friston, 2011). Effective connectivity allows determining the direction of influence between brain elements because it is based on dynamic activity models that take into account information, such as the anatomical connections between neural units and the simultaneous interaction of the elements within the model (Friston, 2011).

The effects of aging on brain connectivity have been previously reviewed for several cognitive functions in task-related and resting-state studies (Antonenko and Flöel, 2014; Sala-Llonch et al., 2015); thus, these reviews discuss brain connectivity in aging related to cognition in general. Two reviews have focused on brain connectivity associated with episodic memory. One of these reviews (Palacio and Cardenas, 2019) included task-related and resting-state studies performed in healthy adults between 18 and 65 years old. However, no distinction was made regarding participants' age, and brain connectivity results in task-related and resting-state studies were discussed jointly without distinguishing their different contributions to episodic memory. The other review (Jeong et al., 2015) covered studies in task-related and resting-state conditions with healthy young adults and adults affected by different pathologies, such as Alzheimer's disease or mild cognitive impairment; however, only one positron emission tomography (PET) study in healthy older adults was included. Therefore, no previous review has exclusively analyzed the effects of aging on brain connectivity during episodic memory, particularly during recollection.

Numerous episodic memory fMRI studies have identified several brain regions that exhibit activation changes associated with aging. Evidence from these studies has provided valuable information regarding the network that is responsible for encoding and retrieving personal memories and the specific brain regions that may be affected by the aging process. However, little is known about how the connectivity among the brain regions that comprise this episodic memory network is affected by aging. Although various studies on the topic have emerged in the last two decades and their findings have significantly enlightened the subject, their contributions have remained dispersed in the literature. Therefore, the purpose of the present review is to integrate those findings and analyze them jointly to provide a comprehensive view of how brain connectivity within the episodic memory network changes as a consequence of aging.

### Connectivity analysis

Functional connectivity is often measured by means of pairwise correlations between time series activity in specific brain regions. However, when the interest is to analyze the potential connectivity among several brain regions, other methods are more suitable, such as psychophysiological interaction (PPI) or seed partial least square (seed PLS). PPI allows the identification of regions across the whole brain, the activity of which is related to that of a seed region in a specific task, group or experimental condition (Friston, 2011). However, PPI does not provide information about the direction of the connectivity between the seed and the brain regions with which it relates and only provides information about the strength of the regression between their activities (O'Reilly et al., 2012). When the interest is to test the psychological factor for more than two conditions, the generalized psychophysiological interaction (gPPI) offers the possibility of creating a PPI term for each condition in the study (McLaren et al., 2012). Seed PLS is a multivariate analysis that was introduced to the examination of fMRI data by McIntosh et al. (1996). PLS identifies brain regions in the whole brain that interact among each other or with specific seeds or regions of interest (ROIs) within each experimental condition or contrast (Krishnan et al., 2011). To assess functional connectivity, the seed mean signal is correlated with the activity of all the other voxels within each condition and across participants (McIntosh, 1999). PLS generates a latent variable for each condition that indicates the pattern of connectivity with the seed that distinguishes each condition.

Graph theory is a collection of mathematical structures used to describe the organization of brain networks or of any other type of network in nature. The topology of a network model is described by its elements or nodes and by the interaction among these elements or edges (Sporns, 2014). The local or global organization of the network is assessed through descriptive analyses applied to the nodes and edges. To build a brain network, first, the nodes should be defined by dividing the brain into regions mostly based on anatomic or functional principles; then, functional connectivity or edges between nodes are defined by using neuronal time series cross-correlation or any other statistic demonstrating their dependency (Rubinov and Sporns, 2010). However, this interaction does not guarantee that nodes are structurally connected (Zalesky et al., 2012). Graph theory provides some useful network parameters, such as the small-worldness topography organization, which refers to a model that has short paths. Fewer edges are required to connect two nodes and have high clustering, i.e., the degree to which nodes within a cluster are connected (Watts and Strogatz, 1998). These properties allow efficient global and local information transmission within the network (Liao et al., 2017). Another characteristic of a network is its density, which refers to the number of edges with respect to the maximum possible edges that the model can comprise (Liao et al., 2017). In brain networks, density may represent its linking cost to functioning.

Effective connectivity can be measured through structural equation modeling (SEM). SEM is able to examine causality between brain regions and how this influence may vary across experimental conditions or groups. This is achieved because SEM applied to fMRI data takes into account brain regions that are anatomically connected and information about how these regions interact with each other (McIntosh and Gonzalez-Lima, 1994). Then, the covariance or the degree to which the activities between two regions jointly vary is estimated simultaneously with that of other brain regions, i.e., taking into account the influence of other brain regions within the model (McIntosh and Gonzalez-Lima, 1994). Path coefficients represent the direction and connectivity strength between brain regions in the network.

Dynamic causal modeling (DCM), which is another method used to measure effective connectivity, estimates the interaction among brain regions and how they are influenced by experimental conditions. DCM models the rate of change in neural activity relative to time as a consequence of an input signal from an external stimulation or another brain region (Friston et al., 2003). The analysis assumes that the influence that one region exerts over another region occurs after a certain delay (Kahan and Foltynie, 2013); thus, DCM estimates the present state and the immediate future state of a dynamic system. The method consists of specifying plausible realistic models comprised of structurally connected brain regions, the brain region that receives the driving-input or experimental stimulation, and the coupling among regions that are modulated by experimental task conditions. Then, observer data are used to estimate the coupling parameters of the model, i.e., the strength and direction of the interaction among regions. Bayesian model selection is used to select the model that provides the best fit and parsimonious model.

## Method

**Figure 1:**
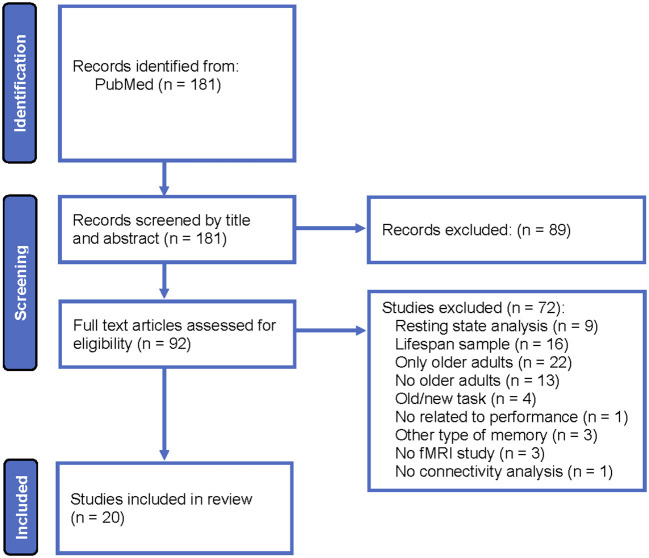
Systematic review flowchart.

A systematic review was conducted according to Preferred Reporting Items for Systematic Reviews and Meta-Analyses (PRISMA) guidelines for all fMRI articles that examined functional or effective connectivity in healthy older adults while participants were performing a recollection task during encoding, retrieval or both phases. Figure 1 displays the systematic review flowchart. All articles were indexed in PubMed, and the publication date was not limited. The search was performed with the following key words: functional magnetic resonance imaging, fMRI, brain connectivity, functional connectivity, effective connectivity, episodic memory, recollection, aging, older adults, and elderly. The results were filtered to exclude studies that included non-healthy adults.

The following eligibility criteria were employed: (i) fMRI studies that included healthy older adults and young adults and (ii) functional or effective connectivity analyses of data recorded during a recollection task. The following exclusion criteria were employed: (i) studies that analyzed brain connectivity during the resting state, (ii) studies in lifespan samples without distinguishing connectivity result differences between young and older adults, (iii) connectivity analyses not related to memory performance, (iv) studies that employed old/new recognition tasks, and (v) studies that did not include young adults. Note that studies that included young, middle-aged, and older adults were incorporated in the current review, but the results obtained in the middle-aged group were not reviewed.

## Results

A total of 20 studies were eligible for inclusion in this review. Table 1 displays the main characteristics of these studies, such as sample size, participants' mean age, and standard deviations when available. The studies in each table were listed in chronological order based on the year of publication. Brain connectivity was analyzed during encoding in five studies, during retrieval in 10 studies and in both phases in five studies. Effective connectivity was analyzed in only four of the studies, and all the rest employed functional connectivity procedures.

**Table 1:** Characteristics of studies included in the review, listed in chronological order.

	**Sample** ***n*****: age (years**, ***M*** ±**SD)**		**Phase**	**Analysis**	
	**Young**	**Older**			
Daselaar et al. (2006)	12: 22.2 ± 2.5	12: 69.2 ± 7.6	Ret	Correlation	FC
Dennis et al. (2008)	14: 19.4 ± 1.3	14: 68.4 ± 7.1	Enc	Correlation	FC
St Jacques et al. (2009)	15: 24.8 ± 4.7	15: 70.2 ± 5.3	Enc	Correlation	FC
Tsukiura et al. (2011)	20: 21.0 ± 3.4	20: 68.6 ± 3.7	Ret	Correlation	FC
Dew et al. (2012)	17: 23.7	14: 66.2	Ret	PPI	FC
Matthäus et al. (2012)	10: 26.3 ± 2.7	10: 67.8 ± 4.0	Ret	Graph theory	FC
St Jacques et al. (2012)	14: 24.4 ± 3.7	14: 64.2 ± 2.9	Ret	DCM	EC
Waring et al. (2013)	19: 23.7	18: 74.4	Enc	SEM	EC
Fandakova et al. (2015)	28: 24.9 ± 1.8	30: 72.3 ± 2.0	Ret	PPI	FC
Grady et al. (2015)	15: 25.6 ± 5.1	15: 69.8 ± 4.7	Ret	Seed PLS	FC
Legon et al. (2016)	14: 26.0	15: 63.0	Enc	DCM	EC
Cansino et al. (2017)	22: 23.4 ± 1.8	22: 67.1 ± 2.7	Enc/Ret	DCM	EC
King et al. (2018)	36: 22.2 ± 3.0	64: 68.4 ± 3.6	Ret	PPI	FC
Monge et al. (2018)	15 19.5 ± 1.3	40: 68.6 ± 6.4	Ret	Graph theory	FC
Ankudowich et al. (2019)	45: 26.1	44: 66.6	Enc/Ret	Seed PLS	FC
Stark et al. (2020)	31: 29.0	31: 76.0	Enc/Ret	Correlation	FC
Varangis et al. (2021)	73: 28.3 ± 4.0	84: 71.2 ± 4.1	Enc/Ret	Graph theory	FC
Deng et al. (2021)	21: 23.5 ± 3.0	20: 70.5 ± 5.4	Ret	Graph theory	FC
Tsuruha and Tsukiura (2021)	29: 22.5 ± 1.7	24: 66.0 ± 3.1	Ret	gPPI	FC
Ness et al. (2022)	48: 26.4 ± 4.2	43: 67.3 ± 5.7	Enc	gPPI	FC

Enc, encoding; Ret, retrieval; PPI, psychophysiological interaction; DCM, dynamic causal models; SEM, structural equation modeling; PLS, partial least squares; gPPI, generalized psychophysiological interaction; FC, functional connectivity; EC, effective connectivity.

Table 2 depicts the type of stimuli employed in each study and the task used during encoding and retrieval. Almost all studies used neutral stimuli, with the exception of three studies that used words or images with positive or negative emotional valence. Recollection was examined using associative tasks in six studies, source memory tasks in six studies and a variety of tasks that included the description of a picture or an event or the imagination of an event, among several others.

**Table 2:** Tasks used in the studies included in the review, listed in chronological order.

	**Stimuli**	**Encoding task**	**Retrieval task**
Daselaar et al. (2006)	Words	Outside the scanner. English word/non-English word	Old/new, confident rate: 1 low/4 high
Dennis et al. (2008)	Faces-scenes	N-back task (*n* = (2)	Definitely old, probably old or new
St Jacques et al. (2009)	Pictures: positive, negative, neutral	Valance rating: positive, negative or neutral	Outside the scanner. Associative: Detail description of the picture after a cue word
Tsukiura et al. (2011)	Faces, names, job titles	Sex judgment of faces	Associative: Selection of the name associated with the face from two learned names, selection of the job title associated with the face from two learned job titles
Dew et al. (2012)	Words	Pleasant/unpleasant Concrete/abstract	Source memory: Select judgment performed in encoding: pleasantness/concreteness
Matthäus et al. (2012)	Words		Personal word or not, then vivid imagination of the cue event
St Jacques et al. (2012)	Words: positive, negative		Retrieve in detail an autobiographic memory trigger by the word, emotion rate:−4 negative/+4 positive, reliving rate: 1 low/8 high
Waring et al. (2013)	Images (positive, negative, neutral)- over neutral scenes	Approach/stay/retreat	Outside the scanner. Old/new for images and scenes presented separately.
Fandakova et al. (2015)	Word-word	Living/nonliving	Continuous recognition: the word-word was presented for the first time (sure new, unsure new) or for the second time (sure old, unsure old)
Grady et al. (2015)	Photographs, cue words		Source memory: Questions about elements from the picture: two options, I do not know
Legon et al. (2016)	Image-image	Detail orientation: anything red? Context orientation: in a kitchen? Response: yes/no	Outside the scanner. Associative: Same or rearranged, yes/no and certainty
Cansino et al. (2017)	Images	Natural/artificial	Source memory: Select the quadrant where each image was presented at encoding
King et al. (2018)	Word-word	Select the object denoted by the words that fit into the other	Associative: Select same pairs, rearranged pairs or new pairs
Monge et al. (2018)	Words	Outside the scanner. Pleasant/unpleasant Bigger/smaller than a shoebox	Source memory: probably pleasantness or size, definitely pleasantness or size
Ankudowich et al. (2019)	Faces	Pleasant/neutral	Source memory: Select the face presented at the left or at the right at encoding, easy task (three face pairs), hard task (six face pairs).
Stark et al. (2020)	Images	Continuous incidental encoding: indoor/outdoor	Repeated, similar and new images No memory task was required.
Varangis et al. (2021)	Words		Logical memory, word order and paired associates
Deng et al. (2021)	Pictures, labels	Label representativeness rate: 1 low/4 high	Associative: Detail recollection of the picture that accompanied the label, memory detail rate: 1 low/4 high
Tsuruha and Tsukiura (2021)	Words	Stimuli were presented in a videoclip by a person belonging to the same age group or different age group	Source memory: Same age group, different age group, new
Ness et al. (2022)	Image-face, image-place	Imagination of an interaction between item-face or face-place, vividness rate: 1 low/4 high	Outside the scanner. Associative: (20 min or six days after encoding; select the face (four options) or the place (four options) that accompanied the image

The contrast used by each study to analyze connectivity is described in Table 3. The main brain connectivity findings obtained in each study are displayed in Table 4. The specific ROIs or seeds used in each study are shown where available. This table depicts whether connectivity or other parameters increased or decreased in older adults relative to young adults. Remarkably, 11 studies used the hippocampus as an ROI or seed, and two more reported findings related to this brain region. The other region that was the second most often used as an ROI or seed or that showed significant connectivity results was the amygdala. This region was used in three studies. Some brain region connectivity analyses were reported more frequently across studies, as noted for the superior parietal cortex and adjacent areas (5 times), superior occipital cortex and adjacent areas (4 times), dorsolateral prefrontal cortex (PFC) (4 times), ventrolateral PFC (3 times), orbitofrontal cortex (3 times), cingulate (3 times), and parahippocampal cortex (3 times). Across studies, connectivity was analyzed among 39 different brain regions that yielded significant results related to aging. However, given that most of the studies included similar regions, a total of 76 brain region connectivity analyses showed significant results associated with the aging process.

**Table 3:** Contrast employed to analyze recollection in each study included in the review, listed in chronological order.

Daselaar et al. (2006)	A quasi- exponential function based on old responses with confident rate 4 vs. old responses with confident rates 1, 2 or 3
Dennis et al. (2008)	Subsequent definitely old responses vs. subsequent likely old and new responses
St Jacques et al. (2009)	Negative pictures subsequently remembered minus subsequently forgotten vs. neutral pictures subsequently remembered minus forgotten
Tsukiura et al. (2011)	Hits during the retrieval of names and job titles vs. misses
Dew et al. (2012)	Context cue trials without memory test vs. correct context trials
Matthäus et al. (2012)	Block design: Time series recorded during episodic memory demands
St Jacques et al. (2012)	Modulatory inputs: 50% more episodic richness than semantic details from verbally retrieved memories from the scanner session within two days later
Waring et al. (2013)	Subsequently remembering item and background vs. subsequently remembering item, for positive and negative scene valences
Fandakova et al. (2015)	Correct rejection of rearranged pairs vs. correct rejection of novel pairs
Grady et al. (2015)	Incorrect and I do not know answers vs. correct answers
Legon et al. (2016)	Modulatory inputs: *F*-contrast over detail and context orientation conditions at encoding
Cansino et al. (2017)	Modulatory inputs: subsequent recollection vs. subsequent unsuccessful recollection; recollection vs. unsuccessful recollection
King et al. (2018)	Hits for intact pairs vs. rearranged pairs judged as intact (false alarms)
Monge et al. (2018)	High confident hits for source memory
Ankudowich et al. (2019)	Orthogonal polynomial contrasts for encoding and retrieval: Retrieval accuracy vs. right PFC vs. left hippocampus
Stark et al. (2020)	Time series across the entire scan, independent of task condition
Varangis et al. (2021)	Time series concatenated from the three episodic memory task
Deng et al. (2021)	High memory based on memory detail rates 3 and 4; low memory based on memory detail rates 1 and 2
Tsuruha and Tsukiura (2021)	Hit source for same age group; Hit source for different age group
Ness et al. (2022)	Subsequent durable memories (6 days after) vs. subsequent transient memories (20 min after)

PFC, prefrontal cortex.

**Table 4:** Brain connectivity results reported in the studies included in the review, listed in chronological order.

	**ROI/seed**	**Results in older adults relative to young adults**
Daselaar et al. (2006)	Hippocampus Rhinal cortex	**↓** Hippocampus**—**Retrosplenial cortex, left parietotemporal cortex **↑** Rhinal cortex—PFC
Dennis et al. (2008)	Left hippocampus	**↓**Occipitotemporal cortex, parahippocampal gyrus, superior parietal, thalamus, hypothalamus, posterior cingulate **↑** Ventrolateral PFC, superior PFC, mid-dorsolateral PFC, orbitofrontal cortex, cingulate gyrus
St Jacques et al. (2009)	Amygdala	**↓** Ventrolateral PFC, left hippocampus **↑** Dorsolateral PFC, fusiform gyrus, posterior parietal cortex
Tsukiura et al. (2011)		**↓** Hippocampus—left anterior temporal lobe
Dew et al. (2012)	Left hippocampus	↓ Red nucleus ↑ Superior temporal gyrus, inferior frontal gyrus, anterior cingulate, precentral gyrus
Matthäus et al. (2012)		↓ Small-worldness, left—right occipital lobes, left—right parietal lobes, hippocampus—amygdala
		↑ Density and size network, left parietal—left frontal regions
St Jacques et al. (2012)	Hippocampus, ventrolateral PFC	↓ Modulation ventrolateral PFC → hippocampus
Waring et al. (2013)	Several ROIs	↑Positive orbitofrontal **↔** dorsolateral PFC, positive orbitofrontal **↔** amygdala, negative amygdala **↔** fusiform, positive fusiform **↔** superior parietal lobe
Fandakova et al. (2015)	Left anterior PFC	↑ Right postcentral, parahippocampal and middle temporal gyri in older adults whose activation patterns deviate less from that of young adults
Grady et al. (2015)	Inferior frontal operculum and anterior insula	= No significant task-related connectivity differences between young and older adults in episodic memory
Legon et al. (2016)	Right hippocampus, visual association area, right inferior frontal gyrus	↓ Modulation of the inferior frontal gyrus → visual association area ↑ Modulation of the inferior frontal gyrus **→** hippocampus
Cansino et al. (2017)	Superior occipital gyrus, hippocampus, orbitofrontal cortex	↑ Modulation superior occipital gyrus **↔** hippocampus **↔** orbitofrontal cortex **↔** superior occipital gyrus during successful encoding and retrieval ↓ Negative driving input of the occipital cortex during successful encoding
King et al. (2018)	Left angular gyrus, medial PFC, left hippocampus, left middle temporal gyrus, posterior cingulate cortex	↓ Average correlations between pairs of anatomical brain regions, except for hippocampus; average correlations between pairs of functional brain regions, except for medial PFC; average connectivity change across all seed and target pairs that exhibited a main effect of age group
Monge et al. (2018)		↑ Widespread functional connections at the medial temporal lobe nodes in the source memory task
Ankudowich et al. (2019)	Right dorsolateral PFC, left hippocampus	↑ Positive hippocampus—bilateral dorsolateral PFC, superior parietal cortex, precuneus, ventral occipital cortex, left inferior parietal cortex, cingulate cortex, negative related to retrieval accuracy
Stark et al. (2020)	Six hippocampal segmentations, parahippocampal cortex, perirhinal cortex, entorhinal cortex	↓ Hippocampus—parahippocampal cortex; three anterior hippocampus regions—parahippocampal cortex
Varangis et al. (2021)		= No significant connectivity metric differences noted between young and older adults in episodic memory
Deng et al. (2021)		↑ Functional integration of PFC with the remainder of the brain network, that was associated with better performance; reconfiguration of connectivity patterns in PFC ↓ Reconfiguration in medial temporal lobe
Tsuruha and Tsukiura (2021)	Right hippocampus, right anterior temporal lobe	↓ Hippocampus, anterior temporal lobe—posterior superior temporal sulcus for words encoded by a person from a different age group
Ness et al. (2022)	Left hippocampus, putamen, caudate	↓ Hippocampus—putamen, caudate for durable memories (six days retention) ↑ Hippocampus—caudate associated with higher durable memory performance

When more than one ROI or seed was used, the specific ROI or seed showing significant results is indicated in the result column.

*PFC*, prefrontal cortex, ↑ increased, ↓ decreased, or = no change in connectivity or other parameters in older adults relative to young adults,—no direction, **→** forward, backward**, or** **↔** bidirectional connectivity.

**Figure 2:**
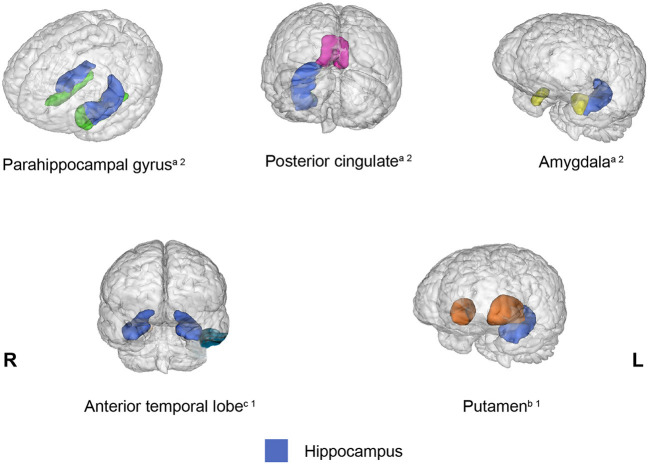
Brain regions that showed decreased connectivity with the hippocampus in older adults relative to young adults across the studies reviewed. When the connectivity was reduced bilaterally, the brain region was displayed in both hemispheres. ^a^Brain regions that exhibited reduced connectivity with the hippocampus during encoding and retrieval. ^b^Brain regions that displayed reduced connectivity with the hippocampus during encoding. ^c^Brain regions that showed reduced connectivity with the hippocampus during retrieval. ^1^One study showed this result. ^2^Two studies showed this result.

**Figure 3:**
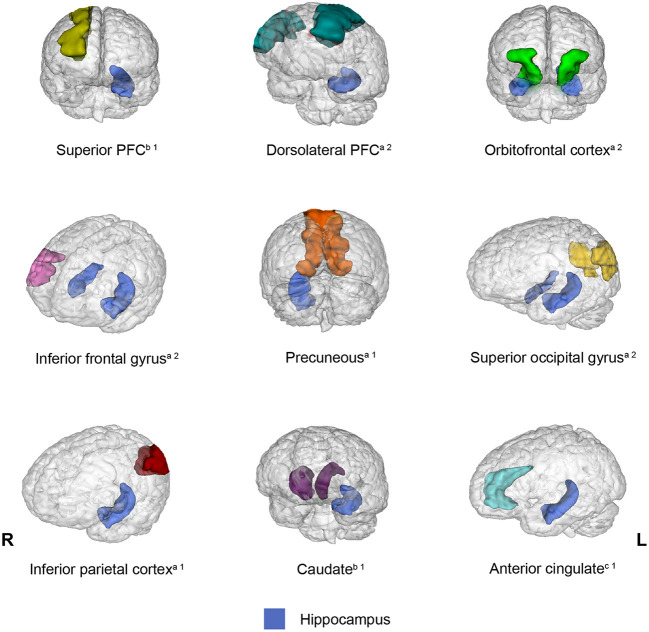
Brain regions that showed increased connectivity with the hippocampus in older adults relative to young adults across the studies reviewed. When the connectivity was increased bilaterally, the brain region was displayed in both hemispheres. ^a^Brain regions that exhibited increased connectivity with the hippocampus during encoding and retrieval. ^b^Brain regions that displayed increased connectivity with the hippocampus during encoding. ^c^Brain regions that showed increased connectivity with the hippocampus during retrieval. ^1^One study showed this result. ^2^Two studies showed this result.

**Figure 4:**
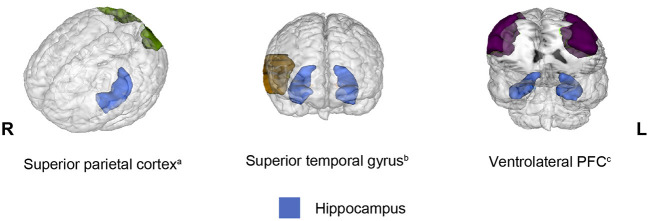
Brain regions that showed opposite results regarding decreased and increased connectivity with the hippocampus in older adults relative to young adults across the studies reviewed. When connectivity was observed bilaterally, the brain region was displayed in both hemispheres. All opposite results involved two studies. ^a^Brain regions that exhibited opposite results during encoding. ^b^Brain regions that displayed opposite results during retrieval. ^c^Brain regions that showed opposite results in different phases.

Connectivity between the hippocampus and several brain regions was observed in 13 out of 20 studies included in the present review. However, a different pattern of connectivity was observed in older adults compared to young adults depending on the brain region with which the hippocampus interacted. To illustrate these findings, Figure 2 shows brain regions (parahippocampal gyrus, posterior cingulate, amygdala, anterior temporal lobe, and putamen) for which connectivity with the hippocampus decreased in older adults relative to young adults. Conversely, Figure 3 depicts brain regions (superior PFC, dorsolateral PFC, orbitofrontal cortex, anterior cingulate, precuneus, superior occipital cortex, inferior parietal cortex, caudate, and inferior frontal gyrus) for which connectivity with the hippocampus increased in older adults compared to young adults. Finally, Figure 4 displays brain regions (superior parietal cortex, superior temporal gyrus, and ventrolateral PFC) for which connectivity with the hippocampus was not consistent. Some studies reported a connectivity increase in older adults relative to young adults, and others reported a connectivity decrease. The brain regions displayed in the figures were identified with the Automated Anatomical Labeling (AAL) atlas (Tzourio-Mazoyer et al., 2002), and the figures were created with the software Mango (ric.uthscsa.edu/mango; Multi-image Analysis GUI).

## Discussion

The relatively few studies that have examined age-related brain connectivity changes while participants attempt to encode or retrieve information from episodic memory by means of recollection processes have provided relevant and consistent information that improves our understanding of the transformations that may occur within the episodic memory network. This notion is particularly true in the case of the hippocampus because its connectivity with several brain regions has been extensively assessed across studies. These results are discussed in detail. Specifically, the mechanism by which the hippocampus modifies its connectivity with other brain regions in older adults compared to young adults is described.

Based mainly on PET and fMRI activation studies, several authors have outlined brain regions that may encompass the episodic memory network. For example, Markowitsch (1995) proposed that the encoding network of episodic memory remains mainly on the hippocampus, cingulate, thalamus, association areas and dorsolateral prefrontal cortex, whereas the retrieval network includes the hippocampal gyrus, medial PFC, orbitofrontal cortex, ventrolateral PFC, and anterolateral temporal cortex. Other authors (Skinner and Fernandes, 2007) proposed that the episodic memory network involved in both familiarity and recollection comprises the medial temporal lobe, sensory regions, parietal, and prefrontal cortices; however, recollection may be distinguished from familiarity by recruiting more areas within these regions. A specific recollection network has also been proposed that includes the hippocampus, lateral parietal region, parahippocampus, anterior medial PFC, and posterior cingulate (Yonelinas et al., 2005). This network is conceived as a core recollection network that is involved independent of the stimulus modality or the task employed to measured recollection (Rugg and Vilberg, 2013).

The brain networks described are highly coincident and clearly illustrate the brain regions that have been more consistently identified as supporting episodic memory. However, brain connectivity analyses, especially those that freely allow the discovery of brain regions interacting across the whole brain, have identified additional regions involved in recollection. Thus, the findings from these studies expand our current knowledge of the regions underlying recollection and especially of those regions in which connectivity is altered by aging. These additional regions are outlined in the following discussion. Given that the effects of aging on brain connectivity could manifest as a connectivity decrease between brain regions or other network parameters or as a connectivity increase between brain regions or other parameters, the following discussion is divided into those brain regions that showed a connectivity decrease, increase or both with the hippocampus as a consequence of aging.

### Decreased connectivity

Activation fMRI studies have frequently interpreted underactivation in healthy older adults compared to young adults as evidence that older adults are unable to allocate sufficient activity from brain regions relevant for the task (Logan et al., 2002), probably due to the employment of other strategies. However, multiple interpretations are conceivable for an observed connectivity decrease in older adults relative to young adults. Certainly, low connectivity between brain regions indicates less transmission of information. However, this reduction in connectivity may be noted because the information has not been processed to a level that is appropriate for transmission and therefore remains at the original brain region to be further processed. Another possibility is that unfeasible information was transmitted that was unsuitable for processing by the receptive brain region. Another alternative is that the region responsible for transmitting the information did not receive the appropriate information in the first place. It is also possible that the information was transmitted but did not reach the appropriate brain region. Another option is that a functional reorganization has occurred, and other brain regions are processing the appropriate information. Although it is impossible to disentangle these possibilities with the information at hand, the intention is to acknowledge the diversity of reasons why brain connectivity may fail.

The hippocampus is certainly the most crucial brain region that supports episodic memory, as demonstrated in several lesion studies in humans and other species and in fMRI experiments (for a review see Squire et al., 2004). Therefore, the hippocampus may be conceived as the key component that operates within the widespread episodic memory network that gives rise to our ability to remember our own experiences. The reviewed studies found that the connectivity between the hippocampus and the following regions diminished in older adults: parahippocampal gyrus (Dennis et al., 2008; Stark et al., 2020) posterior cingulate (Daselaar et al., 2006; Dennis et al., 2008), amygdala (St Jacques et al., 2009; Matthäus et al., 2012), anterior temporal lobe (Tsukiura et al., 2011), and putamen (Ness et al., 2022). All these regions have been included in at least one of the networks reviewed above, except for the amygdala and putamen. The retrosplenial cortex, which also exhibits reduced connectivity with the hippocampus in older adults relative to young adults (Daselaar et al., 2006), is often combined with the posterior cingulate cortex because both regions are sensitive to the amount of information recollected (Rugg and Vilberg, 2013).

All these regions share a common feature. Specifically, these regions share a relative anatomical proximity to the hippocampus without any tendency to be in any particular location relative to the hippocampus, such as anterior or posterior. Moreover, the structural connection between the hippocampus and all these regions has been confirmed using superresolution diffusion MRI (Maller et al., 2019). The parahippocampal cortex is an essential element of the recollection network because it is involved in encoding and retrieving contextual information (Diana et al., 2007). The content of recollection representations is initially sustained by the posterior cingulate and then is transmitted to frontal areas to receive further monitoring and examination (Yonelinas et al., 2005). The role of the anterior temporal lobe in recollection is more controversial because damage to this region has been associated not only with retrograde amnesia but also with semantic memory impairment (Markowitsch, 1995). Although the amygdala has not been conceived as a component of the recollection network, extensive evidence has confirmed that the amygdala modulates memory consolidation (McGaugh, 2004) but not exclusively episodic memory. The putamen contributes to motor execution and motor learning as well as episodic memory, as revealed by several recent studies (for a review see Ell et al., 2011). Specifically, it has been suggested that the putamen interacts with the hippocampus during episodic memory formation.

Reduced connectivity between the hippocampus and the parahippocampal cortex (Dennis et al., 2008; Stark et al., 2020), posterior cingulate (Daselaar et al., 2006; Dennis et al., 2008), and amygdala (St Jacques et al., 2009; Matthäus et al., 2012) was observed during encoding and retrieval. Connectivity reduction between the hippocampus and anterior temporal lobe was observed during retrieval (Tsukiura et al., 2011), whereas connectivity reduction between the hippocampus and the putamen was observed during encoding (Ness et al., 2022). However, because these two last findings were examined in only one phase, either during encoding or retrieval, it is unknown whether this result is exclusive to one of the phases or both. The parahippocampal and posterior cingulate cortices are probably the most relevant regions that showed decreased connectivity with the hippocampus because they belong to the core recollection network. Levels of activation in the hippocampus, parahippocampal gyrus, and posterior cingulate were reported in only one of the studies reviewed (Dennis et al., 2008). Lower activation in older adults than in young adults was observed in the first two regions but not in the posterior cingulate. Thus, the decreased connectivity between the hippocampus and the parahippocampal gyrus may be attributed to the fact that the information was not processed to an optimal level to be transmitted.

According to Eichenbaum et al. (2007), contextual information is formed and represented in the parahippocampal cortex during encoding. Then, this information is transmitted to the hippocampus, where item and context are integrated for episodic representation. In contrast, during retrieval, the hippocampus mediates the recollection of the contextual representation allocated in the parahippocampal cortex. Therefore, the interaction between the hippocampus and parahippocampal cortex is crucial to encode and retrieve episodic representations, and the reduced connectivity between both regions clearly provides an explanation for the gradual loss of recollection in aging. The posterior cingulate is a relay area between the hippocampus and other cortical areas, such as the caudate, orbitofrontal cortex, and anterior cingulate cortex (Pearson et al., 2011). Posterior cingulate activity has been frequently identified during retrieval (e.g., Maddock et al., 2001) but also supports memory consolidation, as observed by its high degree of reinstatement activity. This finding was revealed in a study (Bird et al., 2015) in which posterior cingulate cortex activity was similar during video projection and silent video rehearsal, and this activity was related to the subsequent number of details retrieved one week later. These findings indicate the importance of the interaction between the hippocampus and posterior cingulate for recollection.

### Increased connectivity

Interpreting brain overactivation in older adults relative to young adults represents a challenge in fMRI studies. Overactivation has been frequently construed as the engagement of activity from additional brain regions, not necessarily specialized to the task at hand, to compensate for neurofunctional age decline (Cabeza et al., 2002) or due to the selection of inappropriate brain regions (Buckner and Logan, 2002). A similar difficulty represents the interpretation of increased connectivity in older adults relative to young adults. Although high connectivity between regions indicates that more information is transmitted, several other explanations are possible. For example, one possibility could be that the original region has not received or processed the appropriate information; therefore, this region engenders the increase in connectivity in search of support among other regions. However, another possibility could be that spurious connectivity within the network increased the transmission of useless information that is not suitable for processing.

The studies reviewed revealed that older adults showed increased connectivity between the hippocampus and the following regions compared to young adults: the superior PFC (Dennis et al., 2008), dorsolateral PFC (Dennis et al., 2008; Ankudowich et al., 2019), orbitofrontal cortex (Dennis et al., 2008; Cansino et al., 2017), inferior frontal gyrus (Dew et al., 2012; Legon et al., 2016), inferior parietal cortex (Ankudowich et al., 2019), anterior cingulate (Dew et al., 2012), caudate (Ness et al., 2022), superior occipital cortex (Cansino et al., 2017; Ankudowich et al., 2019), and precuneus (Ankudowich et al., 2019). Interestingly, four frontal areas increased their connectivity with the hippocampus, whereas older adults attempted to recover episodic memories. However, the hippocampus also enhanced its connectivity during recollection with widespread regions. Importantly, except for the dorsolateral PFC and the orbitofrontal cortex, the remaining regions are not conceived as part of an episodic network. However, one of the networks described (Skinner and Fernandes, 2007) made reference to extensive brain areas that may include some of these regions. All these regions are structurally connected to the hippocampus (Goldman-Rakic et al., 1984; Barbas and Blatt, 1995; Catani et al., 2003; Maller et al., 2019).

During encoding, the dorsolateral PFC is engaged in the organization of information before it is encoded. During retrieval, this region is involved in monitoring and verifying the information that has been retrieved (Simons and Spiers, 2003). The orbitofrontal cortex is essential in learning associative information (Duarte et al., 2010). Lesion of the orbitofrontal cortex impairs the ability to recall contextual details of personal experiences, leading to confabulation symptoms (Gilboa et al., 2006). In healthy individuals, confabulation and context misattributions have been attributed to a lack of strategies to control recollection by constraining the memory search to the most plausible cues and retrieval routines (Burgess and Shallice, 1996), and the implementation of these strategies depends on the orbitofrontal cortex (Fischer et al., 1995). The superior PFC is activated during encoding and retrieval according to several studies (e.g., Ranganath et al., 2000); however, its specific role in episodic memory has not been elucidated. The inferior frontal gyrus activity is associated with the attempt to integrate retrieved information into rich episodic contextual associations (Lundstrom et al., 2005), and seems to control the degree of emotion enhancement when recollecting autobiographic memories (Denkova et al., 2013).

Compared to young adults, older adults showed increased connectivity between the hippocampus and the dorsolateral PFC (Dennis et al., 2008; Ankudowich et al., 2019), orbitofrontal cortex (Dennis et al., 2008; Cansino et al., 2017), and inferior frontal gyrus (Dew et al., 2012; Legon et al., 2016) during encoding and retrieval and with the superior PFC (Dennis et al., 2008) only during encoding (the only phase in which it was assessed). Although the superior PFC and the inferior frontal gyrus are not considered components of the episodic memory network, several studies provide evidence that these regions participate during recollection when the brain attempts to retrieve episodic information and when it succeeds (for a meta-analysis see Hasegawa et al., 1999). The participation of these regions during encoding may be determined by the type of processes engaged in encoding. For example, the inferior frontal cortex showed subsequent memory effects when participants performed a semantic living/nonliving judgment of words but not when they performed a phonological task, i.e., odd/even number of syllables (Otten, 2001). Damage restricted to the superior frontal gyrus impairs working memory (du Boisgueheneuc et al., 2006), and it has been demonstrated that working memory contributes to the integration of episodic representations during encoding (Plancher et al., 2018). Moreover, the brain regions that participate during encoding are also expected to participate during retrieval according to the transfer-appropriate processing principle, which assumes that memory recovery depends on the degree to which the original processes used during encoding are present during recovery (for a review see Roediger and Gallo, 2002). Thus, the studies reviewed indicate that the hippocampus interacts with regions engaged to encode and retrieve episodic information to a greater extent in older adults than in young adults.

The hippocampus also increased its connectivity with the precuneus (Ankudowich et al., 2019), and superior occipital gyrus (Cansino et al., 2017; Ankudowich et al., 2019) in older adults relative to young adults. Two regions that were not considered part of the recollection network. However, as occurs for frontal regions engaging in specific processes during encoding and retrieval, recollection also depends on brain regions specialized on the type of information, such as its modality or content. Moreover, according to cortical reinstatement, the regions involved in the encoding of such information are expected to also be activated during retrieval. Indeed, increased connectivity between the hippocampus and the precuneus and superior occipital gyrus in older adults was observed in both phases, providing support for cortical reinstatement.

Three other regions that do not belong to the recollection network also exhibited enhanced connectivity with the hippocampus in older adults compared to young adults: the anterior cingulate (Dew et al., 2012), inferior parietal cortex (Ankudowich et al., 2019), and caudate (Ness et al., 2022). The fact that it is possible to recuperate remote memories after hippocampal damage leads to the conception that the hippocampus is the initial depository of new episodic memories, but because the hippocampus constantly coordinates the reactivation of the cortical network involved in the storage of the specific features that integrate the experience, gradual memories will depend less on the hippocampus and eventually would be supported only by the cortical network (Squire and Wixtede, 2011). The anterior cingulate cortex has been identified as part of this network responsible for memory consolidation and recall of remote memories, according to several lesion studies in rodents Weible et al. (2013). The inferior parietal cortex seems to be responsible for previous mental conditions that may contribute to memory formation, such as maintaining attention on task goals and encoding salient events (for a review see Singh-Curry and Husain, 2009). Additionally, the anatomical connectivity between the hippocampus and the inferior parietal cortex has been interpreted as evidence that the former induces spatial processing in the inferior parietal cortex for memory purposes (Clower et al., 2001). World memory champions, who demonstrate outstanding performance in several memory tasks, showed high volume correlations between the hippocampus and caudate, and both volumes were positively correlated with memory performance (Müller et al., 2018). Moreover, dementia symptoms are associated with the gradual loss of projections from dopaminergic neurons into the caudate (Rinne et al., 1989). However, caudate functions are not exclusively related to memory and contribute to the selection of appropriate actions or strategies to achieve several goals (Grahn et al., 2008).

Increased connectivity between the hippocampus and the inferior parietal cortex was observed during encoding and retrieval (Ankudowich et al., 2019). However, connectivity with the caudate was reported only during encoding (Ness et al., 2022), and connectivity with the anterior cingulate was observed only during retrieval (Dew et al., 2012). However, whether these interaction enhancements exclusively occur in one phase in older adults cannot be excluded until these interactions are examined in both phases. It is crucial to understand the reason for incremental age-related connectivity between the hippocampus and several regions across the brain. One possibility could be that the hippocampus increased its connectivity within the recollection network and beyond as a compensatory mechanism (Cabeza et al., 2002). Notably, this strategy is not sufficient for older adults to compensate for their recollection deficit because their performance was below that of young adults in almost all the studies reviewed, except for studies that found no differences (Grady et al., 2015), controlled task difficulty (Daselaar et al., 2006) or did not report these data (Matthäus et al., 2012; Fandakova et al., 2015; Varangis et al., 2021). However, the lower recollection performance in older adults relative to young adults does not exclude the possibility that connectivity was enhanced as a compensatory mechanism because it is unknown whether the performance would be even lower if connectivity within the network had not increased. Another possibility could be that the hippocampus in older adults interacts with brain regions that are not properly specialized in recollection in accordance with the dedifferentiation hypothesis (Li et al., 2001). However, based on the above discussion, all the regions with which the hippocampus increased its interaction contribute in varied forms to recollection, indicating that these regions may not be conceived as inappropriate to support dedifferentiation. A more plausible explanation could be that the aging brain experiences a recollection network reorganization that adapts to the exigencies of the task at hand during encoding and retrieval by increasing the hippocampus connectivity with specific brain regions. In addition, the hippocampus also undergoes a connectivity reduction with regions conceived as essential for recollection, such as the parahippocampal cortex and the posterior cingulate.

### Decreased and increased connectivity

Three regions showed opposite results across studies (i.e., either decreased or increased connectivity with the hippocampus): the superior parietal cortex (Dennis et al., 2008; Ankudowich et al., 2019), superior temporal gyrus (Dew et al., 2012; Tsuruha and Tsukiura, 2021), and ventrolateral PFC (Dennis et al., 2008; St Jacques et al., 2012); these three regions have structural connections with the hippocampus (Barbas and Blatt, 1995; Kiernan, 2012; Maller et al., 2019). Among these regions, only the ventrolateral PFC has been considered an element of the episodic memory network, particularly during retrieval. This notion is supported by various lines of evidence. Specifically, patients with damage in this area suffer from retrograde amnesia, and PET studies showed activation in this area during retrieval (Markowitsch, 1995). More recently, with the employment of repetitive transcranial magnetic stimulation (rTMS), it was observed that the direct perturbation of the ventrolateral PFC impaired the ability to retrieve details of a previous experience (Wais et al., 2018). Although the empirical evidence has confirmed that the ventrolateral PFC contributes to recollection, this region might not be conceived as part of the recollection core network because activity in this region has been linked to two broad cognitive mechanisms that contribute not only to episodic memory but also to semantic and working memory. These mechanisms include the control of strategies that guide the search for the most suitable memories and the selection of the most appropriate memory when several are retrieved (Badre and Wagner, 2007). Due to the diverse processes attributed to the ventrolateral PFC, the opposite connectivity observed between the hippocampus and this region, which seems to depend on the specific requirements of the task at hand, is understandable. Indeed, the low modulation of the ventrolateral PFC over the hippocampus was observed when older adults were required to retrieve details of previous experiences (St Jacques et al., 2012), a quite demanding task.

The superior parietal cortex does not correspond to the so-called lateral parietal cortex, which is located in the angular gyrus and considered part of the recollection network. Numerous studies (for a review, see Shomstein, 2012) have provided evidence that the superior parietal cortex is responsible for top-down attention control to relevant stimuli or features according to observer purposes. Top-down attention mechanisms exhibit opposing functions to bottom-up attention mechanisms that are guided by stimulus salience and depend on the ventral parietal cortex. A similar distinction has been proposed to explain memory retrieval, referring to the dual attentional processes hypothesis (Cabeza, 2008) or attention-to-memory hypothesis (Ciaramelli et al., 2008). According to these proposals, when memory retrieval is difficult and requires more strategies, the superior temporal lobe allocates top-down attentional resources for retrieval strategies. However, when retrieval is automatic and requires no strategies, bottom-up attentional mechanisms mediated by the ventral parietal cortex are activated to recover memories. Thus, the connectivity between the hippocampus and the superior parietal cortex may be indicative of the amount of top-down attentional resources required for the task at hand. The superior temporal gyrus is involved in several functions, such as auditory processing, language and social cognition, but its role in memory remains unknown.

The opposite connectivity observed in older adults between the hippocampus and the ventrolateral PFC (Dennis et al., 2008; St Jacques et al., 2012), superior parietal cortex (Dennis et al., 2008; Ankudowich et al., 2019), and superior temporal gyrus (Dew et al., 2012; Tsuruha and Tsukiura, 2021) may be due to the different experimental conditions employed across studies and highlights that connectivity may not be conceived as a rigid change in one direction that occurs as a consequence of aging. In contrast, it should be expected that older adults would have decreased or increased connectivity among the recollection network and other brain regions according to task demands and the type of information that integrates the memory representation.

### Conclusions and future directions

The studies reviewed provide strong evidence that the hippocampus orchestrates the brain regions that are engaged during the encoding and retrieval of episodic memory representations, as revealed by its connectivity with almost all brain regions that pertain to the recollection network or that intervene when particular task demands require processes that are attributed to specific brain regions. Moreover, the connectivity experiments reviewed disclose a pattern of how the connectivity among this network is affected by the aging process because most of the brain regions that were coupled with the hippocampus showed a consistent connectivity output in older adults, and only three of them presented opposite results.

Aging crucially changes the connectivity pattern of the recollection network, which includes the hippocampus, parahippocampus, posterior cingulate, lateral parietal cortex and medial PFC (Yonelinas et al., 2005; Rugg and Vilberg, 2013). As revealed by the findings noted across studies, the hippocampus decreased its connectivity with the parahippocampal cortex and posterior cingulate in older adults compared to young adults. None of the studies reviewed analyzed the connectivity between the hippocampus and the lateral parietal cortex and the medial PFC. Thus, whether these regions would show a similar connectivity decrease with the hippocampus remains unknown. Assuming that the parahippocampal cortex and posterior cingulate should be involved in any recollection task, their lower interaction with the hippocampus represents a crucial change that may explain the gradual loss of the ability to retrieve details of our experiences as we age.

However, the aging brain does not remain static to this loss of connectivity between the hippocampus and two essential members of the recollection network. To cope with this limitation, the aging brain seems to undergo reorganization of the recollection network that is characterized by increased interactions between the hippocampus and other regions specialized in distinctive recollection functions. In particular, the increased connectivity between the hippocampus and several PFC regions, such as the dorsolateral PFC and orbitofrontal cortex, which clearly function in encoding and retrieval, seems crucial to overcome recollection decline. The hippocampus also exhibits increased connectivity with several other regions located across the whole brain that support a variety of functions involved in recollection. However, the eventual integration of these additional brain regions within the recollection network depends on task demands, stimulus modality and contextual details. For example, one study (Dennis et al., 2008) that used the working memory n-back task to encode pairs of faces and scenes showed increased connectivity between the hippocampus and the dorsolateral PFC in older adults, a region related to the implementation of executive functions in working memory (Levy and Goldman-Rakic, 2000). Another study (Dew et al., 2012) that used a more conceptual task to encode words (pleasant/unpleasant or concrete/abstract judgments) showed increased connectivity between the hippocampus and the inferior frontal gyrus in older adults, a region that is involved when semantic judgments are required (Otten, 2001). Two studies that examined the recollection of spatial context for faces (Ankudowich et al., 2019) or images of common objects (Cansino et al., 2017) revealed increased connectivity between the hippocampus and occipital cortex in older adults compared to young adults.

Therefore, the increased connectivity observed between the hippocampus and several brain regions does not follow a rigid pattern, and the selection of these regions would depend on the task employed to process the initial information during encoding, which is expected to also be involved during retrieval according to the transfer-appropriate processing hypothesis (Kolers, 1973). Additionally, the selection of additional regions would depend on the stimulus modality or type of contextual information encoded, which are also expected to be engaged during retrieval according to the reinstatement hypothesis (e.g., Alvarez and Squire, 1994). Moreover, it is possible that all regions interact simultaneously with the hippocampus because task processing, which is uncharged mainly by PFC areas, and memory content integration require constant information fluency to update the memory representation to be encoded and retrieved. This notion is based on the reentrant signaling that occurs by reciprocal connections between separate neural groups or maps that are believed to support high brain functions (Edelman, 1993).

However, a closer look at the findings observed in the studies reviewed does not actually support drastic network reorganization in which new brain regions across the whole brain, close and distant to the hippocampus, are recruited to interact with the hippocampus and deal with recollection deficits in the aging brain. Radical neuroplasticity changes of this magnitude are not plausible, even during brain injury or learning, in which changes occur mainly in adjacent areas (Nudo, 2013). Instead, these apparently new regions are already participating in encoding and retrieval. The only difference is the magnitude to which the activity and interaction of these regions are required to achieve the task at hand. Therefore, it is proposed that the brains of young adults also recruit those regions that exhibit increased connectivity with the hippocampus in older adults, but the connectivity with these regions is of lesser magnitude than that noted in older adults. In young adults, the processes that occur within each region, and their interaction with the hippocampus is almost negligible or below the threshold given its low intensity. Conversely, older adults require intensive utilization of those processes and more information fluency to accomplish recollection. Although overall memory performance in older adults is reduced compared with young adults, as discussed above, the connectivity analyses performed by the different studies are based on successful recollection performance, indicating that older adults succeed in those trials but at a higher cost.

Indeed, brain activity and brain connectivity differences between young and older adults are merely variances in intensity and do not imply network modifications altering brain region composition. Therefore, it is proposed that the aging brain experiences the need for intensifying processes and connectivity within the recollection network to achieve successful encoding and retrieval of rich and complex memory representation. This notion may be easily identified as brain-intensification-recourse. This mechanism seeks to counteract the natural decrease that characterizes the aging process in several domains but emerges from the need to combat the decreased connectivity observed between the hippocampus and main regions from the recollection network.

Given that most of the studies reviewed employed functional connectivity analysis, the direction of the connectivity between the hippocampus and several brain regions remains an open question. However, the studies that used effective connectivity analysis also provide information on the direction of connectivity; for example, the ventrolateral PFC diminished its modulation of the hippocampus (St Jacques et al., 2012), the inferior frontal gyrus increased its modulation of the hippocampus (Legon et al., 2016), the modulation between the hippocampus, and orbitofrontal cortex exhibited similar modulations, and between the hippocampus and superior occipital cortex increased bidirectionally during encoding and retrieval in older adults (Cansino et al., 2017). This last finding is consistent with the transfer-appropriate processing, and reinstatement hypotheses.

Thus, the specificity of these findings demonstrates the advantage of effective connectivity. Another difficulty encountered with functional connectivity is that it is based exclusively on correlation. Functional connectivity exists when two regions increase their activity above chance, and functional connectivity does not depend on the presence of a structural connection between the regions (Eickhoff and Müller, 2015). Moreover, functional connectivity between two regions may be due to another brain area wherein connectivity with those regions increases their activity (Eickhoff and Müller, 2015). Additionally, technical and biological noise may cause spurious correlations if both regions are influenced by these artifacts (Birn, 2012). Therefore, functional connectivity should be interpreted with caution.

Our knowledge of the neurofunctional changes that occur in the healthy aging brain to overcome recollection decay would benefit from the employment of connectivity analyses that provide more precise information, such as effective connectivity analyses that allow the assessment of couple direction and the presence of reciprocal connectivity. Several important questions remain unanswered. For example, confirming which brain regions belong to the recollection network and establishing the interaction sequence that these regions follow during encoding and retrieval are important future research topics. It would also be of interest to systematically examine the relevance of each region to accomplish recollection during encoding and retrieval. This information would help to identify the regions and functions that require more attention to prevent recollection decline. It is also important to precisely identify brain regions engaged during recollection according to the process involved and memory content. Additionally, it would be important to confirm that the same brain regions are involved independently of participants' age and that only the intensity of the activity and connectivity among regions varies. Of course, the answer to these questions requires the implementation of well-controlled experiments.

## Author contributions

The author confirms being the sole contributor of this work and has approved it for publication.

## Funding

This work was supported by the National Autonomous University of Mexico, General Direction of Academic Personal Affairs (DGAPA) (Grant No. IG300121).

## Conflict of interest

The author declares that the research was conducted in the absence of any commercial or financial relationships that could be construed as a potential conflict of interest.

## Publisher's note

All claims expressed in this article are solely those of the authors and do not necessarily represent those of their affiliated organizations, or those of the publisher, the editors and the reviewers. Any product that may be evaluated in this article, or claim that may be made by its manufacturer, is not guaranteed or endorsed by the publisher.
